# Carbon clusters on substrate surface for graphene growth- theoretical and experimental approach

**DOI:** 10.1038/s41598-022-20078-x

**Published:** 2022-09-22

**Authors:** Satoru Kaneko, Takashi Tokumasu, Manabu Yasui, Masahito Kurouchi, Satomi Tanaka, Chihiro Kato, Shigeo Yasuhara, Tamio Endo, Akifumi Matsuda, Mamoru Yoshimoto, Musa Can, Sumanta Kumar Sahoo, Kripasindhu Sardar, Jyh-Ming Ting, Masahiro Yoshimura

**Affiliations:** 1 National Cheng Kung University, Tainan, 701 Taiwan; 2 Tohoku University, Sendai, Miyagi 980-8577 Japan; 3 Kanagawa Institute of Industrial Science and Technology (KISTEC), Ebina, Kanagawa 243-0435 Japan; 4 Japan Advanced Chemicals, Sagamihara, Kanagawa 252-0243 Japan; 5 Tokyo Institute of Technology, Yokohama, 226-8502 Japan; 6 Istanbul University, 34134 Istanbul, Turkey

**Keywords:** Surface chemistry, Computational methods, Synthesis of graphene

## Abstract

Growth morphology of carbon clusters deposited on different substrates were investigated by theoretical and experimental approach. For theoretical approach, molecular dynamics was employed to evaluate an adsorptive stability of different size of carbon clusters placed on different substrates. The adsorptive stability was estimated by the difference of total energy of supercell designed as carbon cluster placed on a certain crystal plane of substrate. Among the simulations of this study, carbon cluster flatly settled down on the surface of SrTiO$_{3}$(001). The result was experimentally verified with *layer by layer* growth of graphene by pulsed laser deposition in carbon dioxide atmosphere. The absorptive stability can be useful reference for screening substrate for any target material other than graphene.

## Introduction

Since the isolation of single-layer graphene by the scotch tape in 2004, graphene has been prepared by many techniques, chemical vapor deposition (CVD), pulsed laser deposition (PLD), molecular beam epitaxy (MBE). In general, graphene growth requires metal catalysts, and the graphene sheet has to be transferred to insulating materials for device applications. Direct growth of graphene on insulating substrates is great advantage for post-processing. By the choice of substrates, graphene can grow directly on insulators.

Not limited to graphene growth, choice of substrate for a target material is one of important factors for synthesis of high quality film. For oxide films on silicon substrate, Schlom et al.^1^ comprehensively investigate the thermodynamic stability of binary oxides in contact with silicon substrate. Reaction between silicon and binary oxide are considered from the point of view of Gibbs free energy. The paper is very useful to select oxide materials which can directly grow on silicon substrates without undesired silicide phase. Since only thermodynamics is considered to evaluate the stability, this method can not deal with epitaxial growth. Crystal orientations must be under consideration for epitaxial growth evaluation. In previous report, in order to evaluate the crystal orientation of film growth, absorptive stability was estimated on an oxide material on target substrate by using molecular dynamics (MD) simulation^2^.

For carbon materials in this study, absorptive stability was estimated on carbon cluster designed on various surface of substrate as a supercell. The absorptive stability was evaluated on variety of substrates, and carbon was experimentally deposited on those substrates. The evaluation of absorptive stability of carbon cluster on various surface can be great help for the choice of substrate for graphene growth. In this paper, carbon films were also experimentally deposited on the target substrates, and surface morphology was investigated in comparison to the MD simulation.

**Figure 1:**
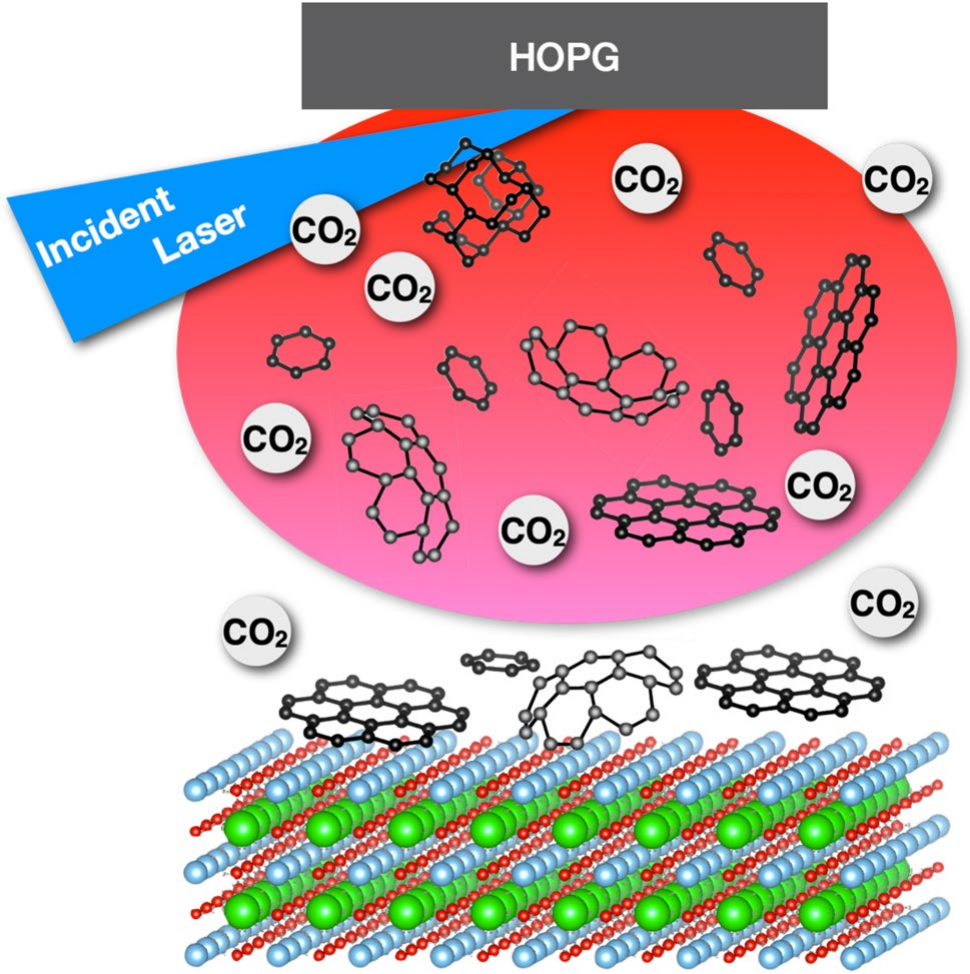
Designing of carbon clusters. With suitable atmospheric environment and choice of substrates, only graphitic carbon clusters can be designed to reach and flatly cover the surface of substrates.

A CVD is one popular method for graphene growth. Carbon films prepared by CVD method are post-annealed in carbon dioxide (CO$_{2}$) instead of strong oxidant oxygen, and show *superflat* surface via selective etching in amorphous carbon^3–5^. Carbon dioxide as a gentle etchant, selectively eliminates intrinsic contamination with post etching process after film growth over 1000 $^{\circ }$C. CVD graphene usually grows with intrinsic contamination on graphene surface during film growth^6^. The graphene surface is simultaneously contaminated with necessary Cu catalyst, where CVD produces active carbon species such as CH$_{n}$ (*n* = 1, 2, 3, ...) and at higher temperature (e.g. 1000 $^{\circ }$C) the carbon species evolve into larger carbon clusters^7,8^. Co-existence of sp2 crystalline carbon (graphitic cluster) and amorphous carbon during a CVD reaction results in a conventional graphene with intrinsic contamination^4^. Since CVD graphene must be transferred onto insulator due to the metal catalyst, as-grown flat graphene on insulators is large advantage for device applications. In this study, we showed a pulsed laser deposition (PLD) grew *superflat* graphene film on a strontium tatanate (SrTiO$_{3}$(001)) which was selected by the MD simulation from candidate materials.

The PLD is a well-known versatile method for preparing a variety of thin films^9–16^ even for preparing nanoparticles^15,17^ and high quality film such as one prepared by using molecular beam epitaxy (MBE)^18^. The PLD irradiates a carbon target and many species of carbon clusters can reach the surface of substrates via a plasma plume, as shown in Fig. 1. In carbon dioxide atmosphere and optimal temperature of substrate, only graphitic carbon cluster can be selectively delivered on substrate surface, and with choice of optimal substrate the graphitic carbon clusters can flatly cover the surface on reaching the substrate. The absorptive stability was estimated to select candidate materials including crystal orientation (crystal plane of substrate).

In previous report, absorptive stability of oxide material was evaluated on silicon substrate, and showed good agreement with experimental results of epitaxial growth^2,19^. In this study, sapphire, magnesium oxide, strontium oxide and silicon were used as candidate materials for evaluation of absorptive stability of carbon clusters. With choice of material including crystal orientation, gas atmosphere and deposition temperature, carbon cluster can be designed as, (1) only graphitic carbon from carbon species in the plasma plume reaches surface of substrate, and (2) the graphitic carbon can flatly cover the surface of the target material.

**Figure 2:**
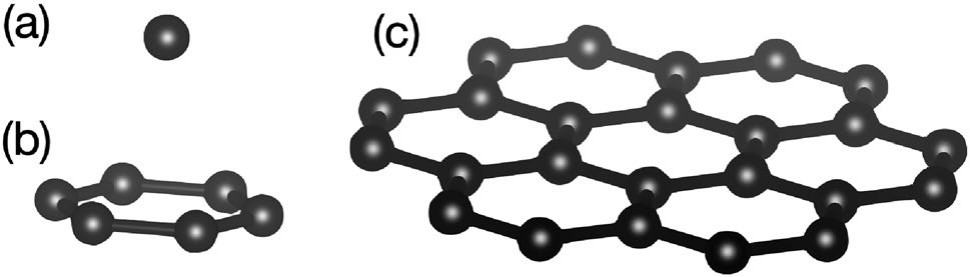
Graphitic carbon cluster. (**a**) a carbon atom (**c**), (**b**) six-membered ring (6-ring) and (**c**) seven six-membered rings (nanographene).

## Experimental methods

Experiments were carried out with combination of theoretical simulations and experimental film growth. For the evaluation of absorptive stability, the molecular model was constructed by Material Studio, and the surface of each substrate was optimized by means of molecular dynamics (MD) with DMol3. The carbon cluster was placed on candidate substrates to estimate absorption energy between the cluster and the substrate surface. A carbon atom (C), six-membered ring (6-ring) and seven six-membered rings (nanographene) were considered as graphitic carbon cluster, as shown in Fig. 2. As an example for strontium titanate (SrTiO$_{3}$), a supercell consisting of 2 $\times$2 $\times$ 2 SrTiO$_{3}$ cells and vacuum region of 20 Å  on the surface as a vacuum slab was used to optimize the surface structure of SrTiO$_{3}$. For absorptive stability, carbon clusters (a C atom, 6-ring and nanographene) were placed in the supercell with the distance of 3 Å  from on the surface as an initial condition. Optimal structure in supercell and the absorption energy were estimated by using the density functional theory (DFT) with a semi-core pseudopotential. The generalized gradient approximation (GGA) method was used to obtain the electron density. The energy difference for self-consistent field was set at 1.0 $\times$ 10 $^{-6}$ Ha (Hartree).

For deposition experiment, a carbon target placed against the substrates was irradiated by the slower Q-switched YAG laser (Spectron Laser System SL803G)^9,20^, as shown in Fig. 1. The target was swung by Python program with Raspberry $\pi$ during the depositions in order to uniformly irradiate the target surface. The carbon target was high oriented pyrolytic graphite (HOPG) and the candidate materials were magnesium oxide (MgO(100)), c-plane sapphire (Al$_{2}$O$_{3}$(0001)), strontium titanate (SrTiO$_{3}$ (001)) and silicon (Si(001), Si(111)) substrate. Table 1 shows the details of deposition conditions. Raman spectra were measured after the film depositions, and atomic force microscopy (AFM) was also employed to observe the film surface, and 2D Fourier transform was used to observe the six-membered carbon ring.

**Table 1:** Conditions for carbon film deposition by PLD. Variety of materials were used as targets in nitrogen, oxygen and carbon dioxide atmosphere with varied distance to the target.

Laser	Wavelength repetition rate fluency	266 nm 2 Hz (slower Q switched) $\sim$ 2 J/cm$^{2}$
Substrate	MgO, Al$_{2}$O$_{3}$, SrTiO$_{3}$, Si	
Gas	N$_{2}$, O$_{2}$, CO$_{2}$	0 $\sim$ 300 mTorr
Deposition	Target distance	20–40 mm
	Substrate temperature	R.T. $\sim$ 800 $^{\circ }$C
	Deposition time	Upto 30 min.

*Slower Q switched YAG^9^.

## Results and discussion

### Choice of substrate

For choice of substrates, absorptive stability was evaluated by supercell consisting of graphitic cluster placed on substrate surface with crystal orientation. Crystal structure of substrate was optimized on supercell with vacuum slab, and the topmost surface was modified by DFT, as shown in *SI Appendix*, Table S1. A carbon atom (C), six-membered ring (6-ring) and seven six-membered ring (nanographene) were used as typical carbon clusters, as shown in Fig 2. Each cluster was optimized, and total energy (E$_{{\mathrm{cluster}}}$) was estimated. The optimized carbon cluster was placed on the surface of strontium titanate (SrTiO$_{3}$), sapphire (Al$_{2}$O$_{3}$), magnesium oxide (MgO) and silicon (Si) substrates, and after optimizing supercell structure, total energy of supercell was estimated (E$_{{\mathrm{supercell}}}$). Absorption energy, E$_{{\mathrm{absorp}}}$, was estimated by

$$\begin{aligned} E_{{\mathrm{absorp}}}= E_{{\mathrm{sub}}} + E_{{\mathrm{cluster}}} - E_{{\mathrm{supercell}}}. \end{aligned}$$

**Figure 3:**
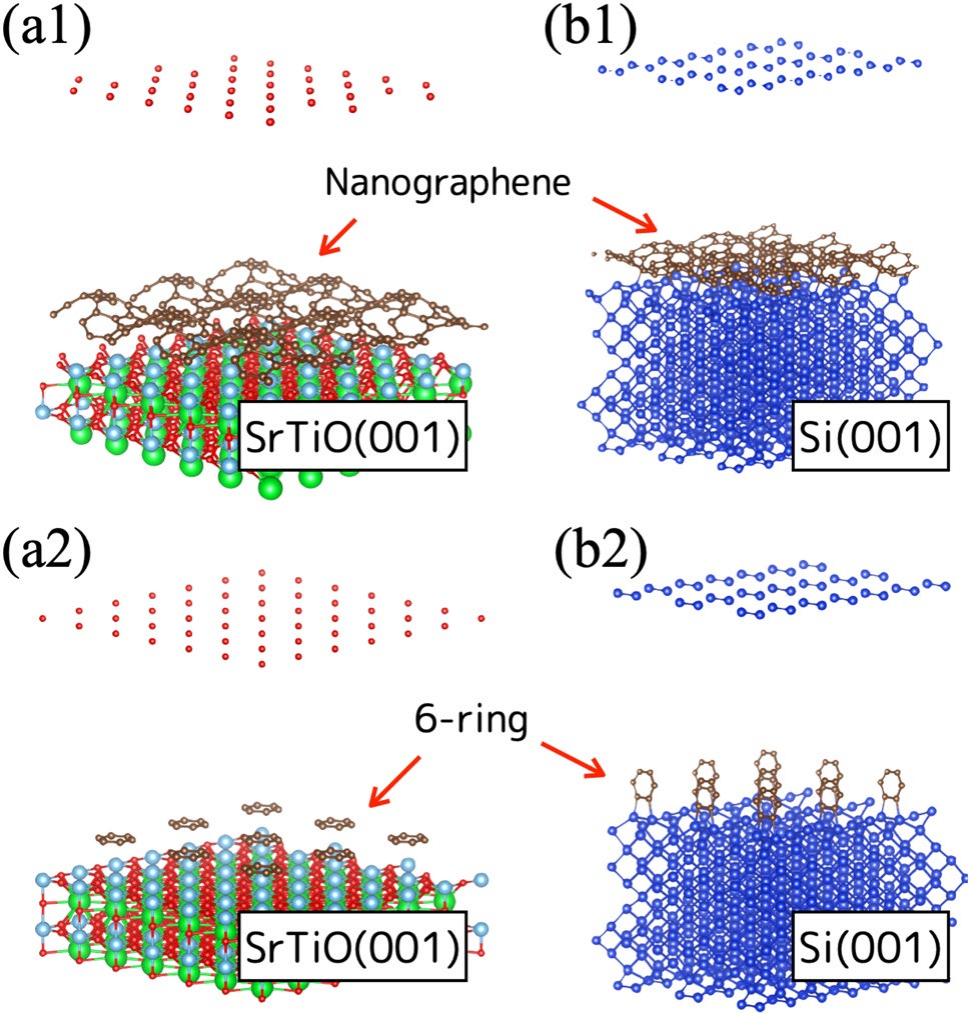
Structure of supercell after optimization. Nano graphene was placed flatly on (**a1**) SrTiO$_{3}$(001) and (**b1**) Si(001) substrates. 6-ring was also placed on (**a2**) SrTiO$_{3}$(001) and (**b2**) Si(001) substrates. The 6-ring stood up vertically on Si surface after optimization while 6-ring flatly lied on SrTiO$_{3}$ surface.

The carbon cluster was placed on either Sr, Ti or O atom on SrTiO$_{3}$(001) surface as an initial condition. The evaluated absorption energies are shown in Table 2. Figure 3 shows schematics of optimal supercell of carbon cluster (nanographene) placed on (a1) SrTiO$_{3}$(001) and (b1) Si(001) surface. Both supercell showed bent surface of nanographene. The absorption energy of nanographene placed on SrTiO$_{3}$ showed $\sim$ 570 kJ/mol on the Sr, Ti and O atoms. On Al$_{2}$O$_{3}$ surface, absorption energy was estimated to be 950 kJ/mol in nanographene placed on either aluminum or oxygen atom. The adsorption energies were almost the same on each atom, and independent of where nanographene was placed on the surface. Since nanographene can be flatly distributed on the surface of SrTiO$_{3}$, Al$_{2}$O$_{3}$ and MgO, graphene seems to grow on those surfaces.

On the other hand, the 6-ring optimized by the DFT stood vertically from Si(001) surface, as shown in Fig. 3b2, while 6-ring was flatly placed on SrTiO$_{3}$(001) surface (Fig. 3a2). The 6-ring was predicted to stand vertically from surface when the 6-ring placed at oxygen atom on Al$_{2}$O$_{3}$, Mg atom on MgO, Si atom on Si(001) and (111). Nanographene might lie flatly on the Si surface, however 6-ring standing vertically from the surface can impede the growth of flat graphene. The DFT optimization of 6-ring resulted in vertically erect 6-ring on Al$_{2}$O$_{3}$, MgO, Si substrate, and flatly folded on SrTiO$_{3}$(001) surface. Both nanographene and 6-ring were expected to lie flat on only SrTiO$_{3}$(001) surface. Graphitic clusters both of 6-ring and nanographene can be designed flatly on only SrTiO$_{3}$ (001) substrate.

### Species of carbon clusters

It is well known that many species of clusters are generated in ablated plasma plumes by PLD^21,22^ and large carbon clusters (e.g. C$_{500}$) are observed in high vacuum atmosphere^23^. Carbon number changes with laser fluency and atmospheric pressure, and clusters with elevated intensity are observed at a range of sizes from few to dozens of molecules. The spectrum of the plume generated from carbon target consists of the C$_{n}$ ions with n = 1, 3, 5, 7, 11, 15 and the number of large cluster decreases with increasing laser fluency in high-vacuum conditions^21^. Energy fluency used for a PLD method usually satisfies the above conditions. The existence of cyclic C$_{6}$ is predicted theoretically^24^ and observed experimentally^25^. Since PLD does not involve carbon hydrate like CVD, PLD has an advantage for intrinsic contamination related to CVD. For the evaluation of absorptive stability, a carbon atom (C), six-membered ring (6-ring) and seven six-membered rings (nanographene) were considered as graphitic carbon clusters, and placed on candidate substrates for the MD simulation.

Catalytic growth of CVD graphene forms contaminated surface with amorphous carbon because of co-exisitance of graphene and intrinsic contamination caused by stability of amorphous carbon in CVD condition. *Superflat* surface of the CVD graphene is observed only after post-annealing in selective etching environment of carbon dioxide at 500 $^{\circ }$C^3^. Graphitic carbon can be supplied in the atmosphere of carbon dioxide over 500 $^{\circ }$C. Since the PLD is most flexible system, deposition atmosphere can be in high vacuum with a variety of gas and a wide range of pressure.

### Choice of atmospheric gas

Oxygen is the most common gas used in plasma cleaning technology such as cleaning surface and ashing photoresist. Carbon films were prepared on sapphire substrates by PLD method in either nitrogen and oxygen gas atmosphere. Raman spectroscopy with the exciting laser wavelength of 785 nm was employed to observe G and D Raman peaks, corresponding to $\sim$ 1550 and 1350 cm$^{-1}$, respectively. Raman spectra from film deposited in nitrogen showed two broad peaks like diamond-like carbon (DLC). Replacing nitrogen with oxygen gas resulted in graphitic film that had strong G and D peaks on Raman spectra by reaction of oxygen on amorphous carbon (*SI Appendix*, Fig. S2).

**Table 2:** Absorption energy estimated on various substrates.

Substrate	Position	Total energy			Absorption energy	Remarks	
		Substrate	Nano graphene	Supercell		Nano graphene	6-ring
SrTiO$_{3}$(001)	Sr atom	− 7321022.51	− 2397450.62	− 9719043.04	569.92	Bent	Slightly rotated
	Ti atom			− 9719042.77	569.65	Bent	
	O atom			− 9719042.49	569.36	Bent	
Al$_{2}$O3(001)	Al atom	− 89556972.78		− 91955383.64	949.56		
	O atom			− 91955383.64	960.25		Vertical standup
MgO(001)	Mg atom	− 23131793.52		− 25530067.18	823.03	Slightly bent	Vertical standup
	O atom			− 25530069.13	824.99	Slightly bent	Lean on O atom
Si(001)	Si atom	− 14880041.41		− 148278044.88	552.85.	Hemisphere	Vertical standup
	Interatomic			− 148278429.79	937.75	Hemisphere	Bent
Si(111)	Si atom	− 18234692.32		− 18833907.40	496.58	Distortion	Vertical standup
	Interatomic			− 18834031.90	621.08	Distortion	Bent

Total energy was calculated on individual substrate (E$_{sub}$) and 7 six membered ring (E$_{graphene}$), one six membered ring (E$_{ring}$), and combined system consisting of substrate and graphene, 7 six membered ring and six membered ring and one carbon atom. Absorption energy was estimated by subtracting of all individual materials and combined system.

Carbon films deposited in nitrogen atmosphere were crumpled in view of optical microscopy, just after being taken out of vacuum chamber due to compressive stress, as shown in *SI Appendix*, Fig. S3. The film thickness of carbon film deposited for 30 min. was more than 2 $\mu$m instead of a few hundred angstrom in oxygen with the same deposition time. In nitrogen atmosphere, almost all clusters stimulated by incident laser beam, including amorphous carbon, reached the substrate surface with no reaction to nitrogen gas. The graphitic films deposited in oxygen gas atmosphere showed flat surface, however the surface roughness was estimated to be root mean square (RMS) $\sim$ 36 nm, which is not flat enough for graphene growth. Oxygen as an etchant gas might be too strong to grow films with flat surface required for graphene growth.

The *superflat* surface without defects is formed on CVD graphene after the removal of intrinsic contamination by CO$_{2}$ *post* annealing at $\sim$ 500 $^{\circ }$C^3^. Carbon dioxide as a gentle etchant, selectively eliminates intrinsic contamination such as amorphous carbon, as a post etching process. CO$_{2}$ is extremely stable materials, however it can be an oxidant by following the equation,

$$\begin{aligned} \mathrm {C + CO_{2}} \leftrightarrow \mathrm {2 CO + \Delta G}. \end{aligned}$$

The above equation can be found in the paper published in 1864^26^. Atmosphere in a chamber can be an oxidation atmosphere even at 500 $^{\circ }$C in low vacuum pressure (0.01 atm). Zhang et.al. employed density functional theory (DFT) calculation to show the reaction barrier of CO$_{2}$ on defect (2.52 eV) is smaller than on graphene surface (4.76 eV)^3^. Intrinsic contamination or unnecessary clusters can be eliminated by reaction with carbon dioxide, and only graphitic carbon cluster reaches the substrate surface. Graphitic carbon cluster can be designed with the PLD method in carbon dioxide atmosphere, with the condition of temperatures greater than 500 $^{\circ }$C, as show in Fig. 1.

**Figure 4:**
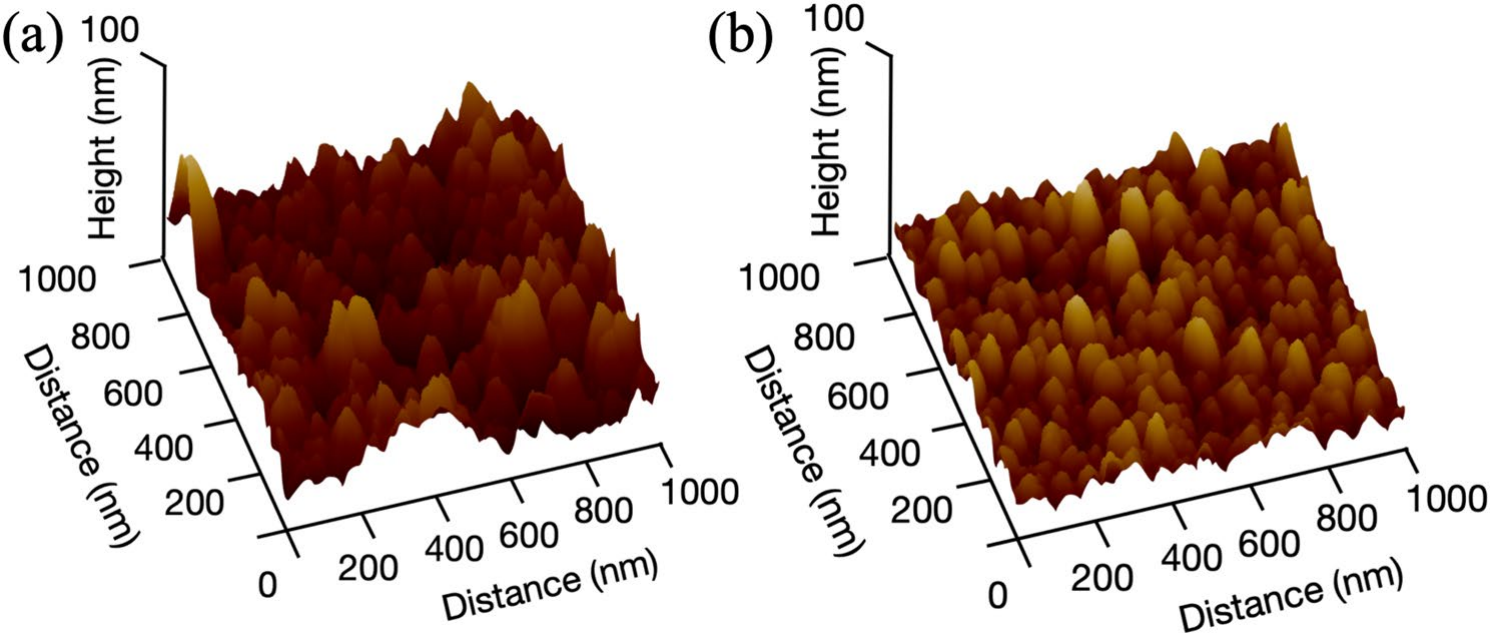
AFM images of surface on carbon films deposited on sapphire substrates in (**a**) oxygen and (**b**) carbon dioxide atmosphere.

The surface roughnesses of graphitic carbon deposited in carbon dioxide was RMS $\sim$ 5 nm on sapphire substrate, instead of 36 nm in oxygen atmosphere, as shown in Fig. 4. Although the film surface prepared in carbon dioxide showed good flatness compared to the one in oxygen atmosphere, the roughness of 5 nm was still rough for graphene growth. In oxygen atmosphere, severe oxidative etching must be dominant and result in rough surface of deposited films. Substrate material and crystal plane should be carefully selected for as-grown *superflat* graphene. For a selection of substrate, in general, many depositions are required on one substrate or another. However, the evaluation of absorptive stability can make it easy to select substrate among many materials with variety of crystal orientation.

**Figure 5:**
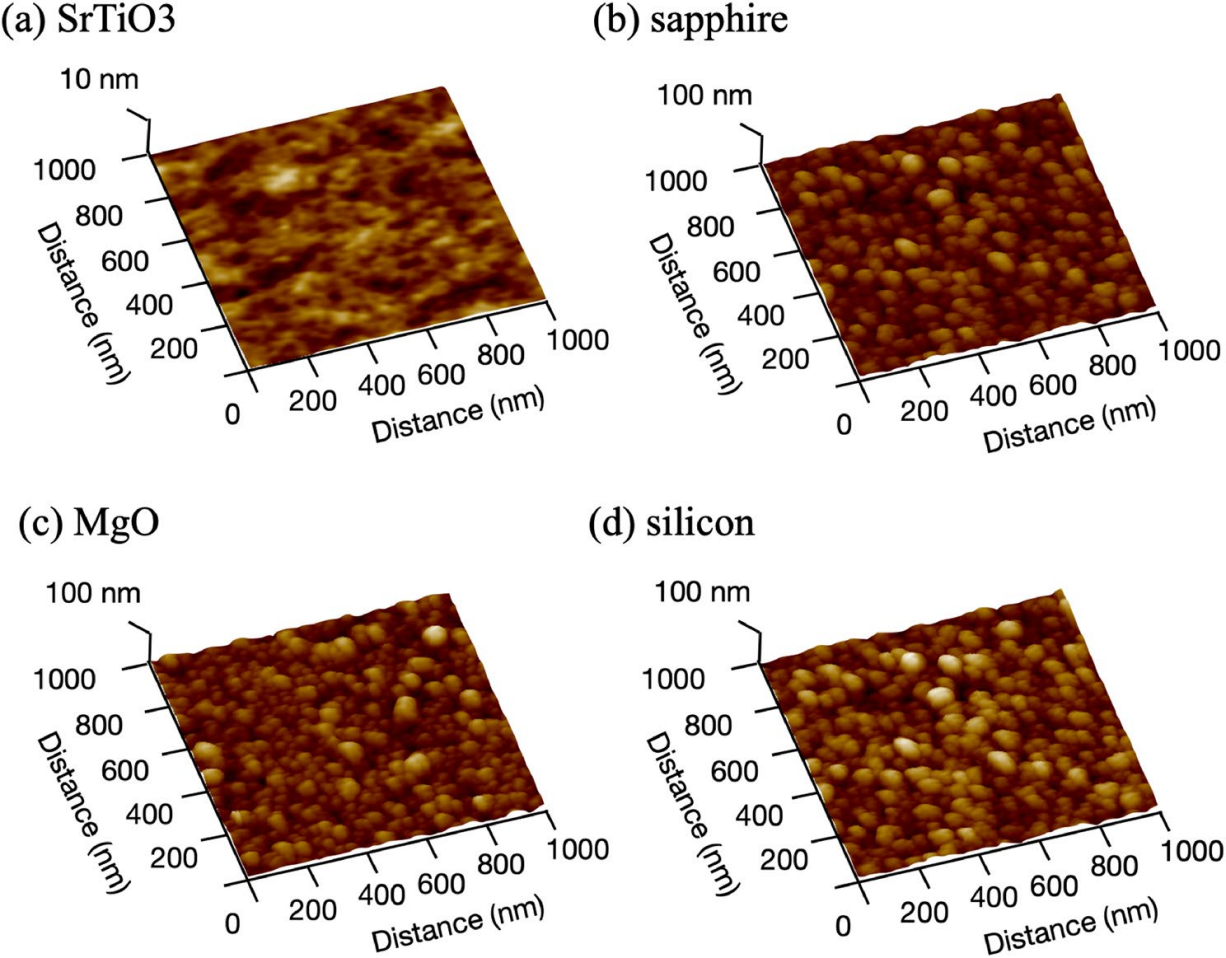
AFM images of film deposited on various substrates; (**a**) strontium titanate (SrTiO$_{3}$(001)), (**b**) sapphire (Al$_{2}$O$_{3}$(001)), (**c**) magnesium oxide (MgO(001)), (**d**) silicon (Si(001)) substrates. Flat surface grew on SrTiO$_{3}$ and nano balls grew on Si substrate.

### Demonstration experiment

**Figure 6:**
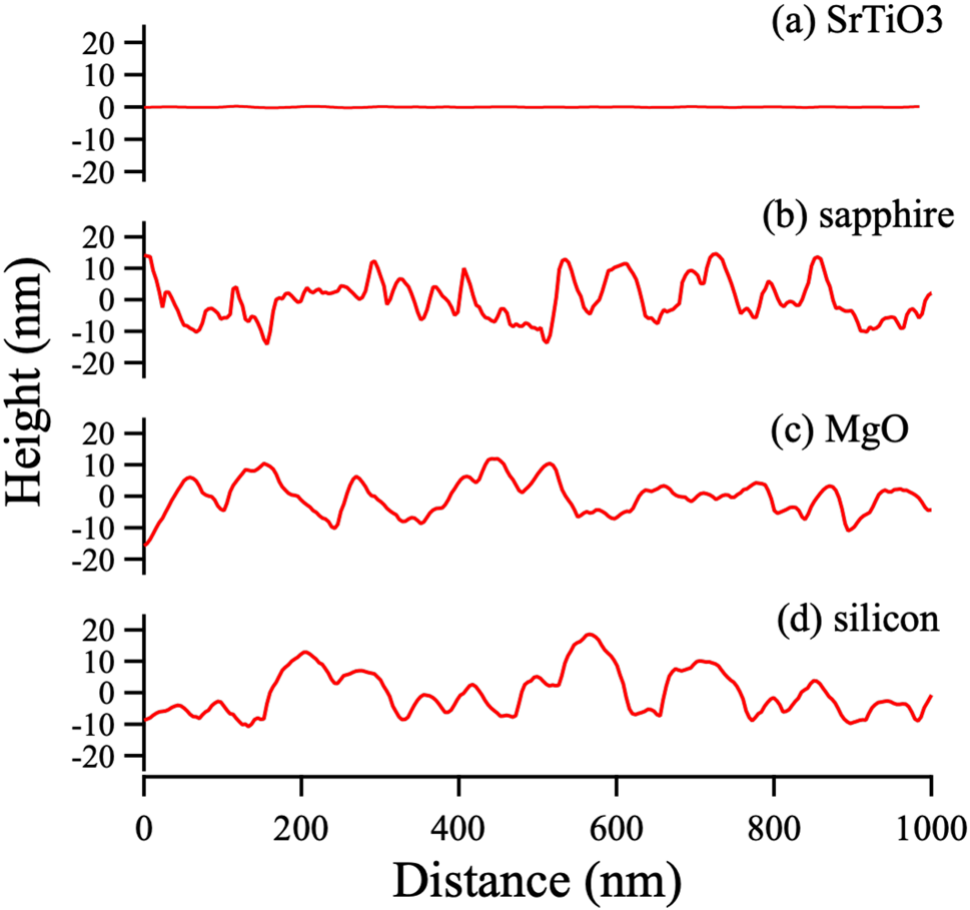
The surface profiles of carbon films on (**a**) strontium titanate (SrTiO$_{3}$(001)), (**b**) sapphire (Al$_{2}$O$_{3}$(001)), (**c**) magnesium oxide (MgO(001)) and (**d**) silicon (Si(001)) substrates.

From the results of the absorptive stability of graphitic clusters, graphene was expected to grow on SrTiO$_{3}$ among the candidate substrates. Variety of substrates were used for carbon depositions in carbon dioxide atmosphere, and the film surfaces were observed by AFM. Figure 5 shows film surfaces deposited on (a) SrTiO$_{3}$(001), (b) Al$_{2}$O$_{3}$(001), (c) MgO(001) and (d) Si(001) substrates by PLD method, and surface profiles are also shown in Fig. 6. As expected, the carbon film showed a flat surface on only SrTiO$_{3}$ substrate, and on silicon substrate the surface morphology was not only coarse and rough, it had the shape of a spheric ball, as shown in Fig. 5d. Yen et al.^27^ reported graphitic nano-ball grows at relatively high temperature and high pressure close to an atmospheric pressure. Interestingly, a cubic MgO grows on Si(001) substrate in high pressure of oxygen atmosphere^17,28^.

As expected by MD simulation, graphene grew *layer by layer* on SrTiO$_{3}$(001) surface. In general, the deposited clusters reach substrate surface and migrate on the surface, and the clusters are captured by kink on the surface resulting in the initial growth of thin film (kink growth)^29^. However graphene grew not from kink but the terrace edge. Graphitic cluster has a 2D structure with covalent bonds, and the cluster was designed to flatly cover the SrTiO$_{3}$(001) surface. The heterostructure growth might be different from the ordinal kink growth model.

All films prepared in carbon dioxide showed relatively flat surfaces compared the ones prepared in oxygen atmosphere, however only film prepared on SrTiO$_{3}$ showed *superflat* surface with RMS $\sim$ 147 pm and Ra $\sim$ 63 pm (*SI Appendix*, S4), which is comparable with *superflat* CVD graphene via CO$_{2}$ annealing (Ra$\sim$76 pm)^3^.

### Graphene as substrate

On the other way around, SrTiO$_{3}$(001) film epitaxially grows on “graphene sheet on SrTiO$_{3}$ substrate”^30^. Since the MD simulation showed both nano graphene and 6-ring lay flat on SrTiO$_{3}$ substrate, it is reasonable that SrTiO$_{3}$(001) film grows on graphene sheet.

Interestingly, magnesium oxide (MgO) also grows on graphene layer^31^ while a metal catalyst is required to grow graphene on MgO substrate^32^. The flat graphene does not grow directly on MgO substrate, which agrees with the results of this study. 6-ring was predicted to vertically stand on the MgO substrate, however nanographene was expected to be placed flatly on the MgO substrate by the MD simulation. When MgO film is deposited on a graphene sheet, nanographene is stable on a MgO surface, so that MgO film is expected to grow on graphene sheet. For the same reason, Al$_{2}$O$_{3}$ and silicon film might grow on the graphene layer, although the graphene did not experimentally grow on those substrates. In fact, Al$_{2}$O$_{3}$ film grows on a graphene sheet^33^. Table 3 shows the summary of graphene growth on each substrate and vice versa.

**Table 3:** The summary of graphene growth on SrTiO$_{3}$, MgO and Al$_{2}$O$_{3}$ substrates and vice versa. (a) graphene film grown on the substrates, and (b) each materials grown on graphene sheet.

(a)		(b)
Graphene film growth		
$\circ$ $^{1}$	SrTiO$_{3}$	$\circ$ $^{2}$
$\times$ $^{1}$	MgO	$\circ$ $^{3}$
$\times$ $^{1}$	Al$_{2}$O$_{3}$	$\circ$ $^{4}$
		Graphene sheet substrate

$$^{1}$$ This work.

$$^{2}$$ Reference^30^.

$$^{3}$$ Reference^31^.

$$^{4}$$ Reference^33^.

## Conclusion

An MD simulation was employed to evaluate the absorptive stability of carbon clusters to select target substrates for graphene growth, and carbon films were experimentally deposited on the targets material in comparison to the theoretical results. MD simulation was employed on sapphire, magnesium oxide, strontium titanate and silicon as target substrates, and the origin of the different morphologies was related to the carbon cluster size. While nanographene flatly covered Si substrate, 6-ring was predicted to stand vertically on the surface. The 6-ring standing vertically from the surface can impede the growth of flat graphene on the surface. Both nanographene and 6-ring were predicted to lie flat on only SrTiO$_{3}$(001) substrate. As expected, as-grown *superflat* graphene was experimentally grown on the SrTiO$_{3}$ substrate. Unlike the thermodynamics stability, the absorptive stability includes crystallographic factors such as crystal orientation of substrates. The absorptive stability can be versatile method to select target substrate with optimal crystal orientation, and together with thermodynamic stability can be great help for material synthesis.

## Supplementary Information

Supplementary Information.

## Data Availability

The datasets generated during and/or analysed during the current study are available from the corresponding author on reasonable request.
