# Supplementary material for: Carbon clusters on substrate surface for graphene growth- theoretical and experimental approach
Source: Sci Rep. 2022 Sep 22;12:15809. doi: 10.1038/s41598-022-20078-x (PMC9500104; doi:10.1038/s41598-022-20078-x)
Supplement: Supplementary file 1 — Supplementary Information. [file 41598_2022_20078_MOESM1_ESM.pdf]

substrate	orientation	slab	atoms	remarks
$SrTiO_3$	(001)	2x2x2	40	4h3m (8 CPU)
Al_2O_3	(001)	2x2x2	241	41h27m (8 CPU)
MgO	(001)	2x2x2	64	1h43 (8 CPU)
Si	(001)	2x2x2	54	4h5m (8 CPU)
Si	(111)	2x2x3	30	2h22m (8 CPU)

TABLE S1 Optimal surface of substrate of strontium titanate, sapphire and silicon. After optimizing crystal surface, a supercell was employed to obtain relaxed surface on each substrate. Remarks show time taken for optimal surface.

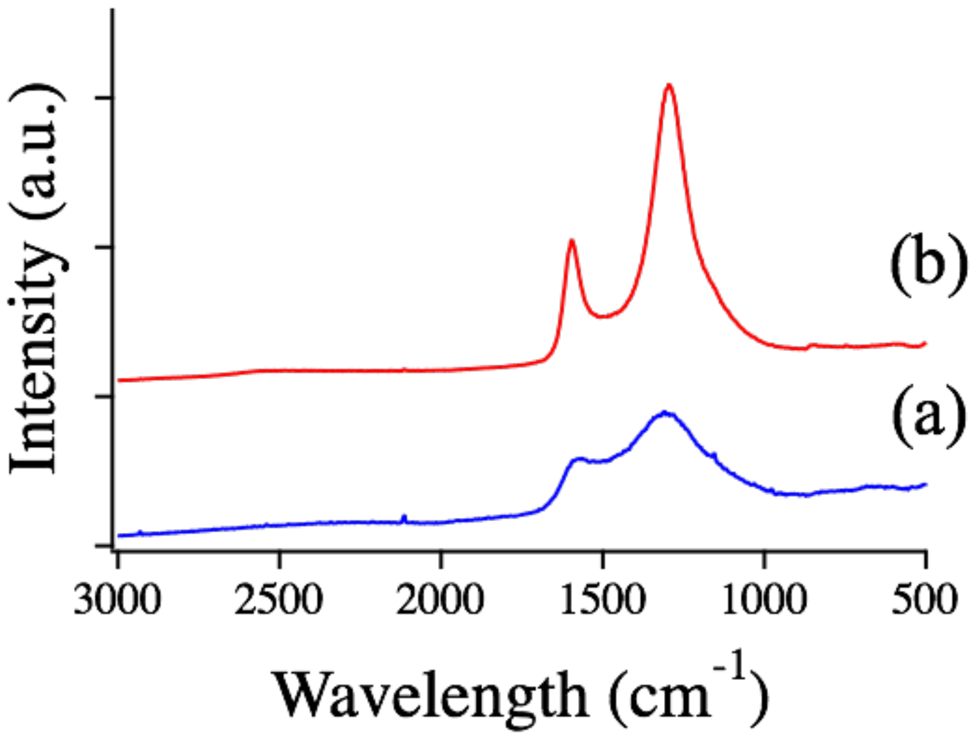

FIG. S1 Raman spectra of carbon film deposited in (a) nitrogen and (b) oxygen atmosphere at the substrate temperature of 800°C. Replacing nitrogen with oxygen results in graphitic film by etching amorphous carbon.

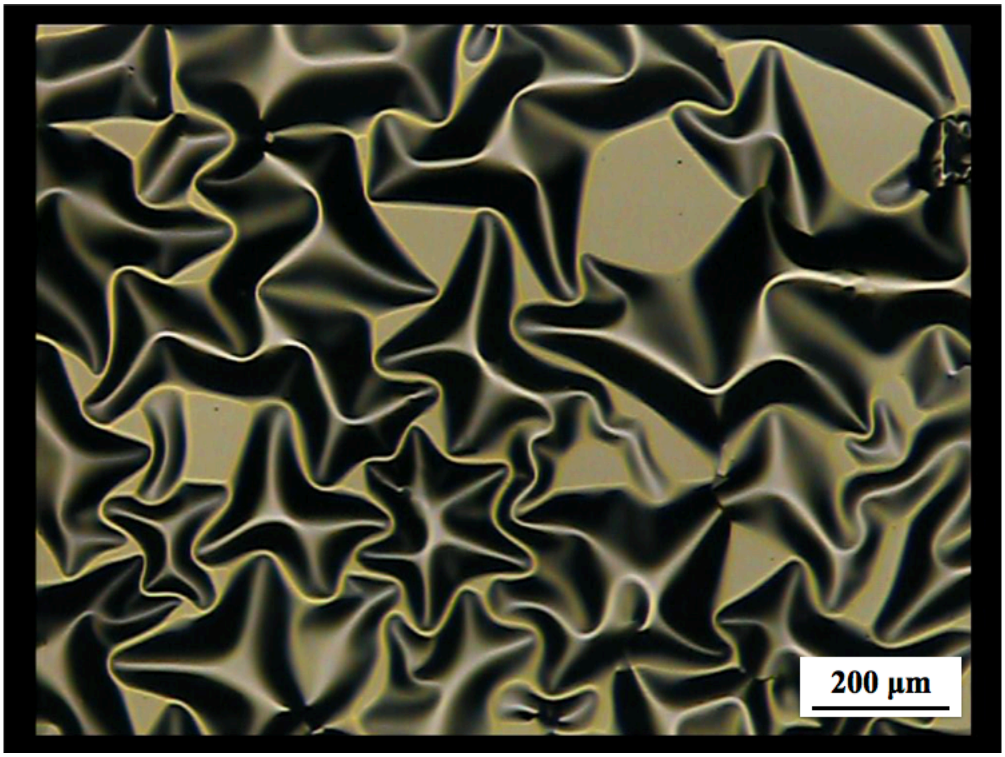

FIG. S2 Photo image of carbon film deposited in nitrogen atmosphere. High deposition rate resulted in thick film thickness (more than 2 μm), and the films were crumpled by compressive stress.

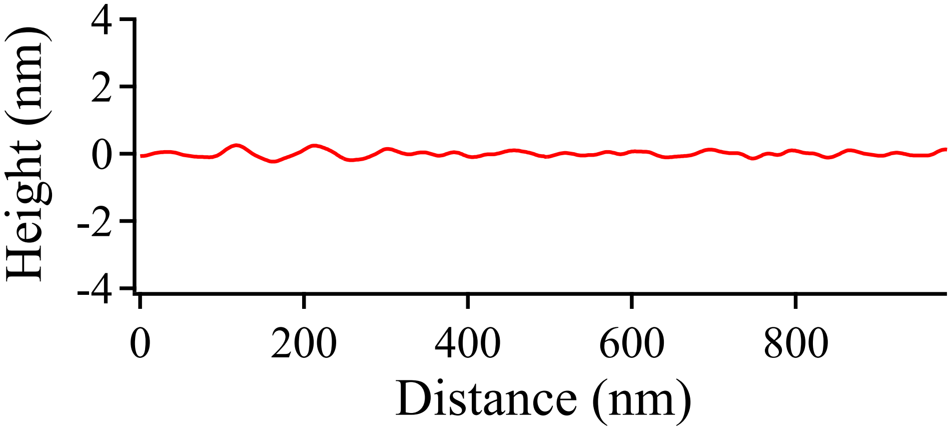

FIG. S3 The roughness on *superclean* surface prepared by PLD in CO_2 atmosphere
